# Retraction Note: A novel, bioactive and antibacterial scaffold based on functionalized graphene oxide with lignin, silk fibroin and ZnO nanoparticles

**DOI:** 10.1038/s41598-025-86520-y

**Published:** 2025-02-04

**Authors:** Reza Eivazzadeh-Keihan, Ensiye Zare-Bakheir, Hooman Aghamirza Moghim Aliabadi, Mostafa Ghafori Gorab, Hossein Ghafuri, Ali Maleki, Hamid Madanchi, Mohammad Mahdavi

**Affiliations:** 1 Catalysts and Organic Synthesis Research Laboratory, Department of Chemistry, Iran University of Science and Technology, Tehran, 16846‑13114 Iran; 2 Protein Chemistry Laboratory, Department of Medical Biotechnology, Biotechnology Research Center, Pasteur Institute of Iran, Tehran, Iran; 3 Advanced Chemistry Studies Lab, Department of Chemistry, K. N. Toosi University of Technology, Tehran, Iran; 4 Department of Biotechnology, School of Medicine, Semnan University of Medical Sciences, Semnan, Iran; 5 Drug Design and Bioinformatics Unit, Department of Medical Biotechnology, Biotechnology Research Center, Pasteur Institute of Iran, Tehran, Iran; 6 Endocrinology and Metabolism Research Center, Endocrinology and Metabolism Clinical Sciences Institute, Tehran University of Medical Sciences, Tehran, Iran

Retraction of: *Scientific Reports* 10.1038/s41598-022-12283-5, published online 24 May 2022

Editors have retracted this Article.

After publication of this Article, concerns about the research it reports were brought to the attention of the Editors. The Editors requested that the Authors provide copies of their ethics approval and protocol for the use of blood samples drawn from a human donor, as well as full raw data, but the Authors were not able to do so.

Hamid Madanchi disagrees with this retraction. The remaining authors did not respond to correspondence from the Editors regarding this retraction.

